# Injective Treatments for Sacroiliac Joint Pain: A Systematic Review and Meta-analysis

**DOI:** 10.1007/s43465-024-01164-w

**Published:** 2024-05-06

**Authors:** A. Ruffilli, T. Cerasoli, F. Barile, M. Manzetti, G. Viroli, M. Traversari, G. Filardo, C. Faldini

**Affiliations:** 1 Department of Biomedical and Neuromotor Science—DIBINEM, University of Bologna, Bologna, Italy; 2 1st Orthopaedic and Traumatologic Clinic, IRCCS Istituto Ortopedico Rizzoli, Via Giulio Cesare Pupilli 1, 40136 Bologna, Italy; 3 Applied and Translational Research Center (ATRc), IRCCS Istituto Ortopedico Rizzoli, Bologna, Italy

**Keywords:** Sacroiliac joint pain, SIJ, SIJ injection, Steroids injection, SIJ steroid injection, PRP

## Abstract

**Background:**

The most effective injective treatment approach for sacroiliac joint (SIJ) pain remains unclear. Aim of this study was to quantify the safety and effectiveness of the available injective strategies to address SIJ pain.

**Methods:**

A systematic review and meta-analysis of the literature was conducted on PubMed, Scopus, and Embase databases from inception until January 2023. Inclusion criteria were studies written in English, comparative and non-comparative studies regardless of the minimum follow-up, and case series on SIJ injections. Safety and efficacy of the different injection therapies for the SIJ were quantified. A meta-analysis was conducted on the available data of the documented injective therapies. The “Checklist for Measuring Quality” by Downs and Black was used to assess the risk of bias and the quality of papers.

**Results:**

The literature search retrieved 43 papers (2431 patients): 16 retrospective case series, 2 retrospective comparative studies, 17 prospective case series, 3 prospective comparative studies, and 5 randomized controlled trials. Of the selected studies, 63% examined the effect of steroid injections, 16% of PRP injections, while 21% reported other heterogeneous treatments. The failure rate was 26% in steroid injections and 14% in PRP injections. The meta-analysis showed a statistically significant reduction in pain with the VAS score for both steroids and PRP: steroids improvement at mid-term 3.4 points (*p* < 0.05), at long-term 3.0 (*p* < 0.05), PRP improvement at mid-term 2.2 (*p* = 0.007), at long-term 2.3 points of the VAS pain scale (*p* = 0.02).

**Conclusions:**

Steroids are the most documented injective approach, with studies showing an overall safety and effectiveness. Still, the high number of failures underlined by some studies suggest the need for alternative procedures. Early PRP data showed promise, but the limitations of the current literature do not allow to clearly define the most suitable injective approach, and further studies are needed to identify the best injective treatment for SIJ patients.

## Introduction

The sacroiliac joint (SIJ) disfunction is a common but underrated cause of low back pain, with 15–30% of patients who complain about low back pain being actually affected by SIJ pain [1, 2]. SIJ can be affected by inflammatory systemic diseases, it can be associated to pregnancy-related changes, it can be the result of failed back surgery [3], and more often osteoarthritis (OA) can also lead to SIJ dysfunction and pain [4, 5]. Regardless of its etiologies, SIJ dysfunction represents a challenge not only for orthopedic surgeons but also for rheumatologists, physical therapists, and osteopaths. The SIJ is involved in sagittal balance [6] and it is crucial in transferring load between the lumbar spine and the lower extremities. Accordingly, SIJ disfunction and pain impairs significantly the quality of life of the affected patients.

Several treatment approaches have been proposed to address SIJ pain. Oral painkillers are the first line of treatment [7], followed by non-interventional physical treatments [8, 9] and non-invasive procedures such as radiofrequency ablations [10]. Surgical management have been proposed for SIJ not responsive to conservative treatments with positive results, but it is an invasive approach affected by a higher rate of complications than non-invasive treatments [11]. In this light, intraarticular injections are gaining increasing interest as minimally invasive treatment. Starting from the use of anesthetics as diagnostic tool [12], the promising results noticed in terms of pain management led to the use of other injective approaches such as corticosteroid injections, prolotherapy with hyperosmolar dextrose, phenol, methylene blue, saline, human growth hormone, botulinum toxin [13–15], platelet-rich plasma (PRP) [16] or autologous bone marrow mesenchymal stem cells (BM-MSCs) [17]. However, a consensus on the most effective treatment for SIJ pain is far from being reached.

The aim of this systematic review and meta-analysis was to quantify the safety and effectiveness of the available injective strategies to address SIJ pain.

## Materials and Methods

A review protocol was established according to the preferred Reporting Items for Systematic Reviews and Meta-Analyses (PRISMA) statement (www.prisma-statement.org) [18]. A systematic literature search was conducted on PubMed, Scopus, and Embase from inception until January 17, 2023. The search string was (“sacroiliac” OR “sacro-iliac”) AND (“inject*” OR “conservative”). No filters were applied. Inclusion criteria were studies written in English, comparative and non-comparative studies regardless of the minimum follow-up, and case series reporting more than five cases of SIJ injections. Preclinical and cadaveric studies were excluded. Studies concerning specific populations such as children, pregnant women, military, high performance athletes, or patients affected by spondyloarthropathy, or other autoimmune diseases were excluded. Safety and efficacy of the different injection therapies for the SIJ were quantified. All studies meeting the inclusion criteria were reported as detailed in Table 1. A meta-analysis was conducted, when possible, based on the available data on the documented injective therapies.

**Table 1:** Details of the included studies

First author	Year	Study design	N° initial patient (treated)	Type of treatment	Injected amount	Final follow-up	Scores	N° failure (definition and timing)	N° complication (and definition)
*Carr C.M*	2016	Retrospective case series	N.R	Anesthetic + steroid	2–3 mL	24 h	Immediate complications	2	10
*Chandrupatla R.S*	2022	Retrospective case series	100	Anesthetic + steroid	N.R	2.6 ± 2.4 months	NRS	60	0
*Plastaras C.T*	2012	Retrospective case series	162	Anesthetic + steroid	2 mL	72 h	Likert pain scale	0	37
*Voelker A*	2022	Prospective case series	N.R	Anesthetic + steroid	N.R	12 weeks	NAS, ODI, CRP, LC	0	25
*Ab Aziz S.N.F*	2022	Prospective case series	31	Anesthetic + steroid	2 mL	6 months	VAS, RMD	16	0
*Liliang P.C*	2009	Prospective case series	39	Anesthetic + steroid	2 mL	45.4 ± 12.0 weeks	VAS modified Oswestry score	13	3
*Aguirre D.A*	2005	Prospective case series	10	Anesthetic + steroid	3 mL	18 ± 2 months	VAS	0	0
*Brändle K*	2016	Prospective case series	35	Anesthetic + steroid	2 mL	2 months	Three-level Likert scale	N.R	N.R
*Bydon M*	2014	Prospective case series	30	Anesthetic + steroid	3 mL	N.R	VAS, 3 level Likert scale, provocative test	4	0
*Medani K*	2021	Retrospective case series	64	Anesthetic + steroid	2 mL	126 ± 94 days	VAS	6	0
*Burcu Duyur Çakit*	2007	Prospective case series	37	Anesthetic + steroid	2.5 mL	1 month	VAS, ODI	21	0
*Andalib A*	2022	Prospective case series	27	Anesthetic + steroid + contrast medium	2 mL	6 months	NRS, Oswestry Disability Questionnaire	4	0
*Cohen S.P*	2021	Retrospective case series	67	Anesthetic + steroid	3 mL	3 months	NRS, Oswestry Disability Score	1	2
*Fouad A.Z*	2021	Prospective case series	34	Anesthetic + steroid	3 mL	1 month	ODI; NRS	3	0
*Fritz J*	2008	Retrospective comparative study	22	Anesthetic + steroid + contrast medium	1.2 mL	12 months	VAS	11	0
*Fritz J*	2008	Retrospective comparative study	35	Anesthetic + contrast medium	1 mL	12 months	VAS	11	0
*Hawkins J*	2009	Retrospective comparative study	155	Anesthetic + steroid + contrast medium	2–2.5 mL	101 months	>50% relief of the targeted pain during the local anesthetic phase and at least 2 weeks of >50% relief afterward	14	0
*Krishnan R*	2021	Retrospective comparative study	104	Anesthetic + steroid + contrast medium	N.R	1 month	VAS	59	0
*Nacey N.C*	2016	Retrospective comparative study	N.R	Anesthetic + steroid	1 mL	1 week	NRS	0	0
*Şahin O*	2021	Prospective case series	46	Anesthetic + steroid	N.R	26.7 months	VAS	5	0
*Savran Sahin B*	2015	Retrospective case series	67	Anesthetic + steroid	N.R	27.8 months	VAS	10	0
*Schneider B.J*	2019	Prospective case series	35	Anesthetic + steroid	2 mL	6 months	ODI, NRS	0	0
*Schneider B.J*	2017	Prospective case series	25	Anesthetic + steroid	2 mL	4 weeks	ODI, NRS	0	0
*Scholten P.M*	2015	Retrospective case series	49	Anesthetic + steroid	2 mL	8 weeks	Provocative maneuver, NRS	0	0
*Siahaan Y.M.T*	2022	Prospective case series	55	Anesthetic + steroid	N.R	9 months	NRS	31	0
*Suleiman Z.A*	2018	Prospective case series	26	Anesthetic + steroid	4 mL	36 months	NRS, ODI	0	0
*Vandervennet W*	2020	Retrospective case series	128	Anesthetic + steroid	3 mL	4 weeks	4-point Likert scale	38	0
*Visser L.H*	2013	RCT	18	Anesthetic + steroid	1.1 mL	12 weeks	VAS	9	0
*Visser L.H*	2013	RCT	15	Physiotherapy	N.A	12 weeks	VAS	12	0
*Visser L.H*	2013	RCT	18	Manual therapy	N.A	12 weeks	VAS	5	0
*Barbieri M*	2022	Prospective case series	10	PRP	2 mL	6 months	VAS, ODI, PGIC	6	0
*Chen A.S*	2022	RCT	15	PRP	2 mL	6 months	ODI, NRS	12	0
*Chen A.S*	2022	RCT	11	Steroid + anesthetic	2 mL	6 months	ODI, NRS	7	0
*Melbourne C.S*	2020	Retrospective case series	15	PRP	N.R	1 year	Questionnaire of perception of outcomes associated with their PRP injection(s)	8	0
*Mohi Eldin M*	2019	Retrospective comparative study	124	PRF	20 mL	6 months	VAS	0	0
*Mohi Eldin M*	2019	Retrospective comparative study	62	PRP	10 mL	6 months	VAS	0	0
*Navani A*	2015	Retrospective case series	10	PRP	4 mL	12 months	VAS, SF-36 PCS, SF-36 MCS	0	0
*Singla V*	2016	RCT	20	LF-PRP	3.5 mL	3 months	VAS, MODQ, SF-12	0	11
*Singla V*	2016	RCT	20	Steroid + anesthetic + saline	3.5 mL	3 months	VAS, MODQ, SF-12	0	4
*Wallace P*	2020	Prospective case series	50	PRP	10 mL	6 months	NRS, ODI	0	0
*Atluri S*	2022	Prospective comparative study	40	N.A	1 mL	12 months	ODI, NRS, EQ-5D-3L, GMH Score, GPH Score	0	0
*Atluri S*	2022	Prospective comparative study	40	BMC	1 mL	12 months	ODI, NRS, EQ-5D-3L, GMH Score, GPH Score	0	0
*Dubick M.N*	2015	Retrospective case series	49	rhGH	10 mL	12 months	ODI, Mankoski pain scale	10	0
*Hoffman M.D*	2018	Retrospective case series	103	Anesthetic + prolotherapy	10 mL	6–7 months	ODI	50	0
*Murakami E*	2018	Prospective case series	85	Anesthetic + contrast medium	0.5 mL	N.R	Groin pain, sitting pain on a chair, SIJ shear test, tenderness of PSIS of STL	13	0
*Dreyfuss P*	2009	RCT	31	Saline	2.5 mL	1 week	Provocative test	0	0
*Dreyfuss P*	2009	RCT	31	Anesthetic	2.5 mL	1 week	Provocative test	0	0
*Murakami E*	2007	Prospective comparative study	25	Anesthetic + contrast medium	1–2.5 mL	N.R	“Restriction of activities of daily life” scoring system from the Japanese Orthopaedic Association	15	0
*Murakami E*	2007	Prospective comparative study	25	Anesthetic + contrast medium	0.5–1 mL	N.R	“Restriction of activities of daily life” scoring system from the Japanese Orthopaedic Association	0	0
*Lee J.H*	2010	Prospective comparative study	20	Botulin toxin + contrast medium	N.R	3 months	NRS, ODI	3	0
*Lee J.H*	2010	Prospective comparative study	19	Anesthetic + steroid + contrast medium	N.R	3 months	NRS, ODI	4	0
*Kim W.M*	2010	RCT	24	Dextrose + anesthetic	2.5 mL	15 months	ODI, NRS	0	1
*Kim W.M*	2010	RCT	26	Steroid + anesthetic	2.5 mL	15 months	ODI, NRS	1	1
*Ward S*	2002	Retrospective case series	10	Phenol + saline	N.R	24 weeks	McGill short-form pain questionnaire	1	3

N.R., non-reported; NRS,; ODI, Oswestry Disability Index; VAS, visual analog scale; RMD, Roland-Morris disability questionnaire; CRP, C-reactive protein; LC, leukocytes; PGIC, Patients’ Global Impression of Change; SF-36 PCS, Short Form Health Survey 36 Physical; SF-36 MCS, Short Form Health Survery 36 Mental; MODQ, Modified Oswestry Disability Questionnaire; SF-12, Short Form Survey 12; EQ-5D-3L, European Quality of Life 5 Dimensions 3 Level version; GMH Score, Global Mental Health; GPH Score, Global Physical Health

### Data Extraction

Two reviewers (FB and TC) screened the articles by title and abstract. The preliminary selection was reported in two tables and continued with the blinded evaluation of full texts. A data extraction form was created using Excel (Microsoft) based on the literature search. The form was filled independently by the two reviewers. At the end of the screening, a full consensus was achieved with the consultation of a third reviewer in case of disagreement. The data extracted included: title, authors, year of publication, journal, type of study, blinding, number of initial patients treated, number of initial SIJ treated, number of patient for final follow-up, number SIJ for final follow-up, age, sex, height, BMI, diagnosis, OA grade, duration of pain pre-treatment, previous treatments, type of treatment, details of treatment, injective approach, radiological guidance, intraarticular or periarticular injection, injection site, injection protocol, injected amount, minimum and final follow-up, scores, failures, and complications.

### Assessment of Risk of Bias and Quality of Evidence

The two reviewers used the “Checklist for Measuring Quality” by Downs and Black to assess the risk of bias and the quality of papers [19]. The checklist consists in 27 ‘yes’ or ‘no’ questions upon 5 sections: 10 items about the overall quality of the study, 3 items about the ability to infer conclusions of the study, 7 items about the study bias, 6 items about selection bias, and 1 item about the power of the study. A third co-author was consulted in case of discrepancies.

### Statistical Analysis

An independent statistician conducted the statistical analysis using Microsoft Excel, following Neyeloff et al. [20]. To produce pooled rates among the studies, the Mantel–Haenszel method was used. The Cochran Q statistic and I2 metric were used as heterogeneity statistical test, a significant heterogeneity was identified by I2 values > 25%; if I2 < 25%, a fixed effect model was used to assess the expected values and 95% confidence intervals. Otherwise, a random effect model was applied, and an I2 metric was calculated for the random effect to test the correction of heterogeneity. The continuity-corrected Wilson intervals were used to calculate the confidence intervals of the studies’ rate.

## Results

**Fig. 1:**
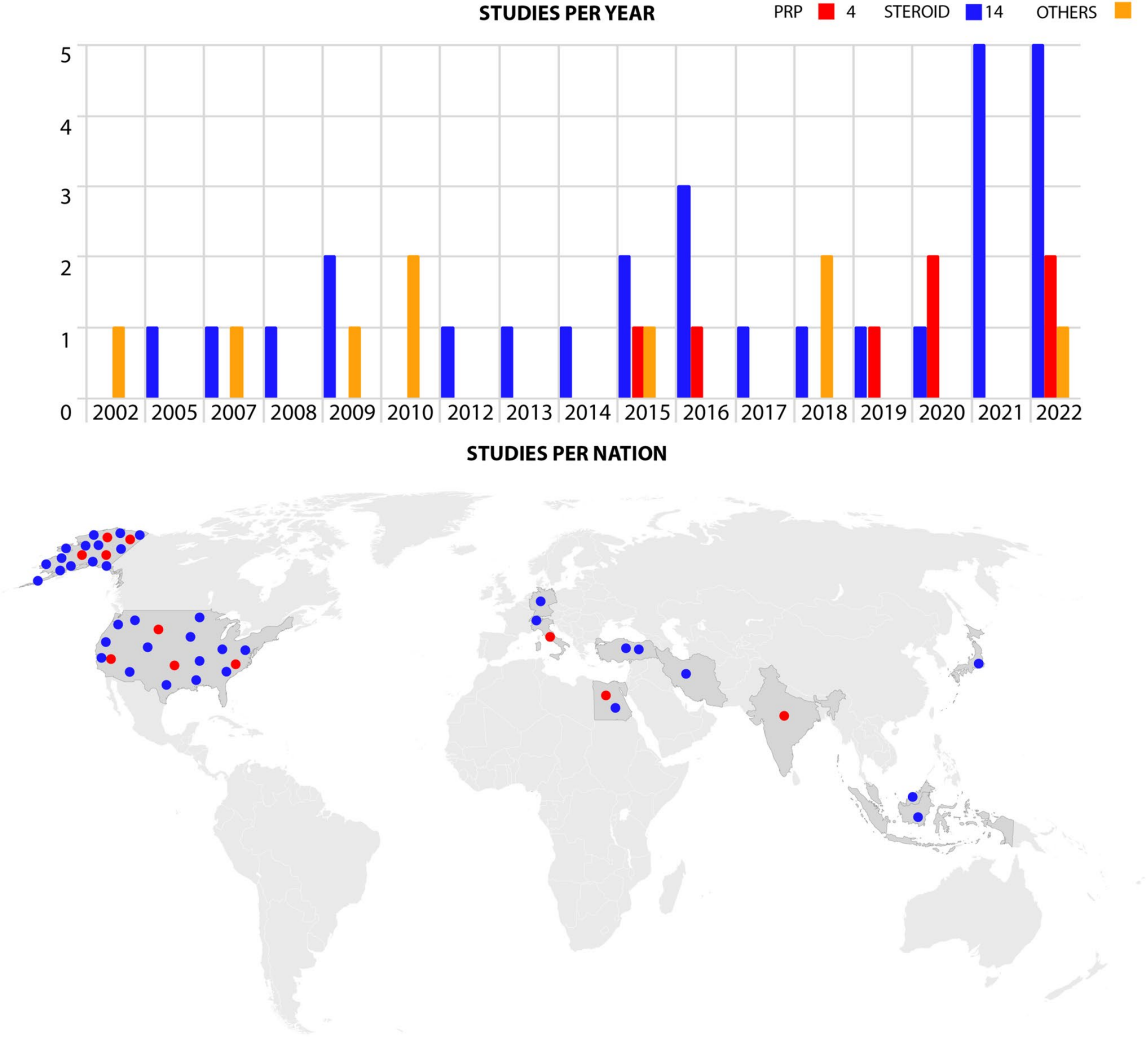
Number of studies per year and geographical distribution of steroid and PRP studies

**Fig. 2:**
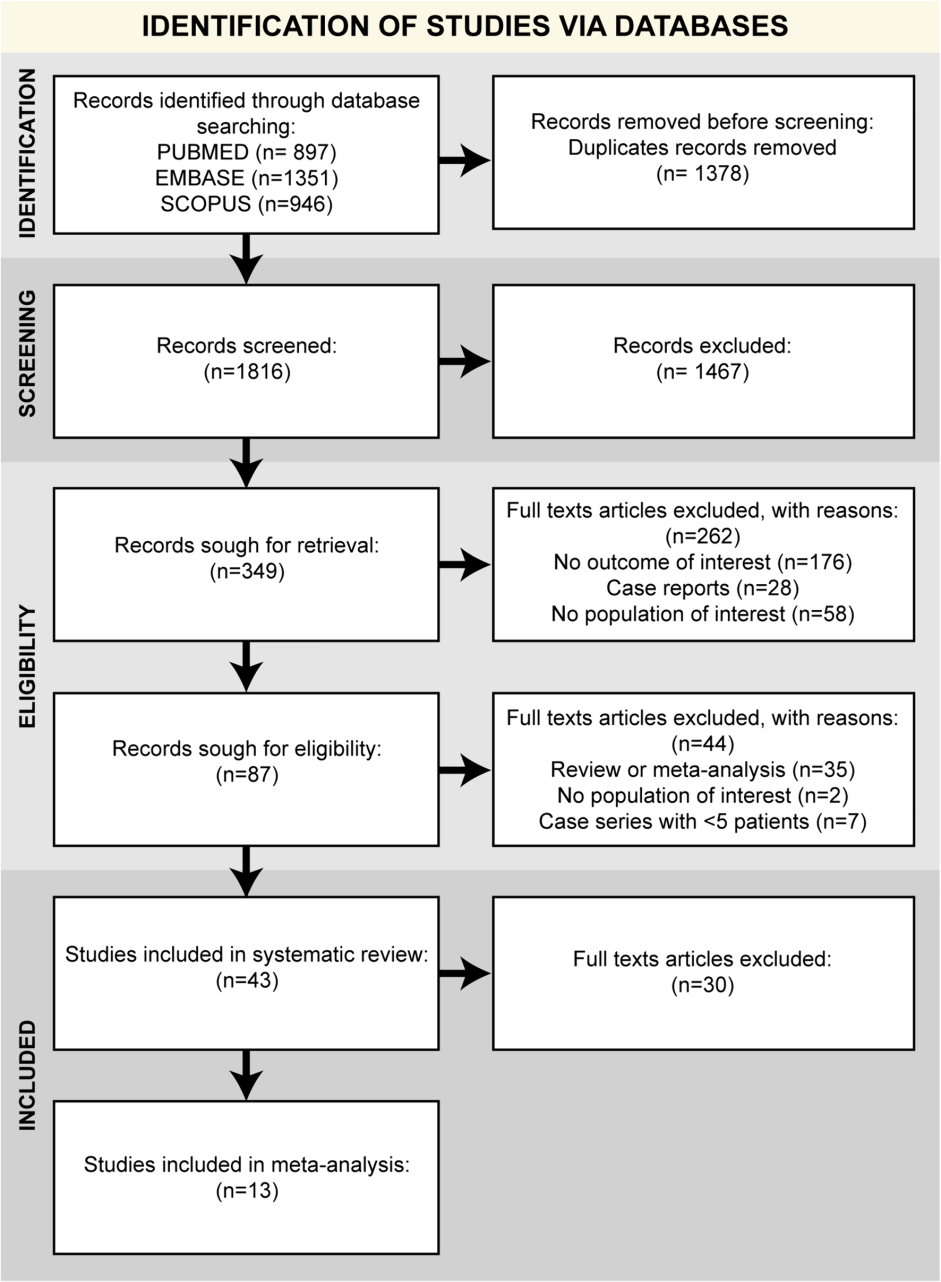
PRISMA flow chart

The literature search retrieved 897 articles in PubMed, 946 articles in Scopus, and 1351 articles in Embase. After duplicates removal, 1816 papers were screened. After full-text screening, 43 papers were selected for the systematic review: 16 retrospective case series, 2 retrospective comparative studies, 17 prospective case series, 3 prospective comparative studies, and 5 randomized controlled trials. The number of studies per year increased over time with 50% of the studies published since 2018 (Fig. 1). Most of the studies were conducted in the USA including 51% of the patients, followed by Japan (9%), Egypt (9%), and Turkey (6%) (Fig. 1). The summary of the selection process is reported in the PRISMA flow chart (Fig. 2) [18].

Of the selected studies, 63% examined the effect of steroid injections, 16% of PRP injections, while 21% reported other heterogeneous treatments. The number of patients retrieved in the systematic review was 2431 (1237 women, 679 men, not reported in the remaining cases), 1425 with steroids, 306 treated with PRP, 700 with other treatments, while 3 studies did not specify the number of patients while only reporting the number of SIJ treated. The mean age of patients treated with steroid injection was 50.4 ± 15.7 years and the mean BMI was 30.2, the mean age of patients treated with PRP was 47.0 ± 15.9 years and the mean BMI was 27.0. The most common guidance used for injection was fluoroscopy (30 studies), followed by computer tomography (CT) (5 studies), ultrasound (4 studies), and anatomical landmarks (2 studies). The injection approach was intraarticular in 33 studies, periarticular in 2 studies, and combined in 4 studies. Among the studies reporting the injection precise localization, 11 were in the lower third and 1 in the middle-lower third. The injection volume ranged from 0.5 to 10 mL. Studies reporting an injection amount of 0.5 mL used anesthetics, studies reporting an injection amount of more than 3 mL used dextrose, rhGH, and PRP, while intermediate volumes were injected when corticosteroids were used.

### Systematic Review Results

**Fig. 3:**
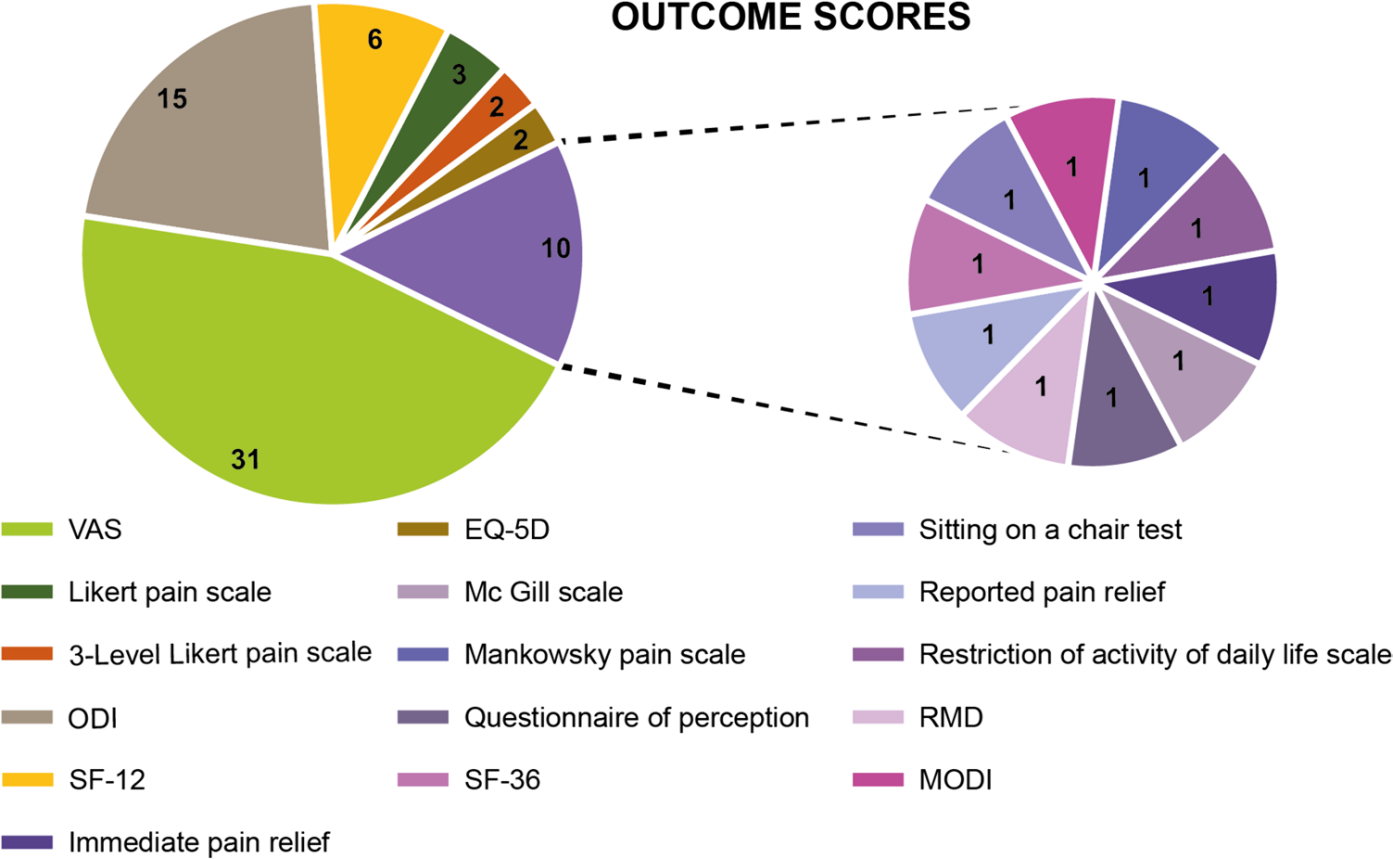
Utilized scores

Out of the 43 studies, one did not analyze failures and complications. Overall, the other 42 studies reported 438 failures and 86 complications. The failure rate was 26% in steroid injections and 14% in PRP injections. Two articles about SIJ steroid injections reported a high rate (19 and 50%) [21, 22] of unspecified complications, while the other studies reported a lower number of complications: overall, the complication rate was 1% in steroid injections and 1% in PRP injections. The used scores were heterogeneous (Fig. 3), with the two most used being VAS and ODI score. No studies described a worsening of the VAS score, while two groups showed a worsening of the ODI score: a control group treated conservatively, and a group treated with prolotherapy (dextrose).

Among the included studies, five were comparative studies. Murakami et al. in 2007 tested the effectiveness of periarticular anesthetic injection for SIJ pain. They suggested to perform first a periarticular fluoroscopy injection and only if the reduction of pain was not significant, another intraarticular injection, highlighting the effectiveness of the periarticular injection and the greater ease of the procedure [23]. Nacey et al. in 2016 confirm the benefits of the periarticular approach, which provided the same results compared with the intraarticular fluoroscopy-guided of steroids [24]. The impact of the choice of steroid for the injection was investigated in 2021 by Khrishnan et al. who did not document any significant difference in outcomes between the groups treated with triamcinolone and methylprednisolone [25]. Anesthetic and steroid injections were compared with botulinum toxin by Lee et al. who suggested in 2010 a greater reduction in pain scores and a longer effect of the botulinum injections [26]. More recently, orthobiologics were investigated for the treatment of SIJ pain. In 2019, Eldin et al. compared platelet-rich plasma (PRP) and platelet-rich fibrin (PRF), with overall promising results and a higher VAS score improvement in patients treated with PRF [27]. Finally, Atluri et al. in 2022 compared the effect of BM-MSCs injections with standard non-interventional therapy, with promising results in favor of BM-MSCs in terms of greater improvement in functional and pain scores [17].

### Meta-analysis Results

**Fig. 4:**
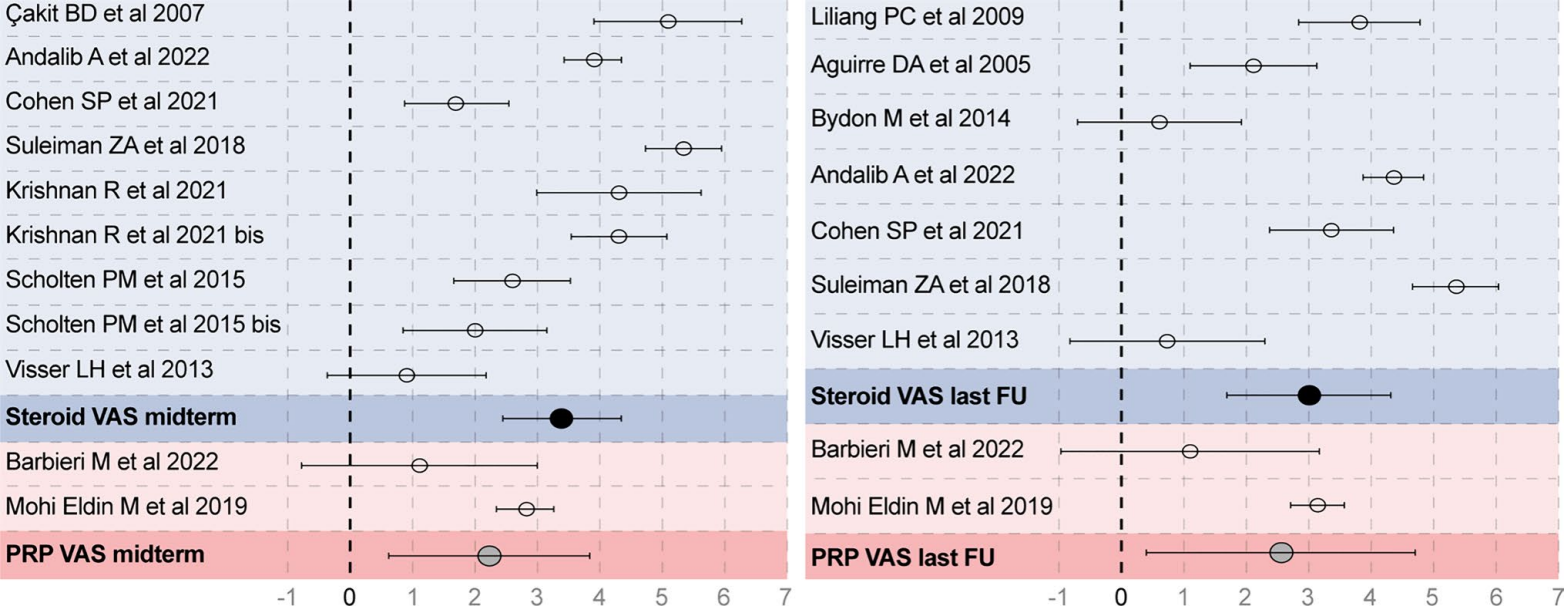
Statistical results and funnel plot

The meta-analysis of the study outcomes was feasible for VAS in 13 studies. The meta-analysis population included 627 patients, 431 treated with steroid injections (11 studies) and 196 with PRP injections (2 studies). The type of steroid injected was *Triamcinolone* *acetonide* in six studies, *Methylprednisolone* in four studies, and *Betamethasone* in one study. VAS scores were separated for early follow-up (from the day after the injection until 1 month after the injection), mid-term follow-up (2–6 months after the injection), and long-term follow-up (>6 months after the injection). The meta-analysis was conducted on VAS at mid-term and long-term follow-up. The reduction in pain recorded with the VAS score was statistically significant in both follow-ups for both steroids and PRP: steroids improvement at mid-term 3.4 points (*p* < 0.05), at long-term 3.0 (*p* < 0.05), PRP improvement at mid-term 2.2 (*p* = 0.007), at long-term 2.3 points of the VAS pain scale (*p* = 0.02) (Fig. 4). Further meta-analysis of the study outcomes was not feasible due to the heterogeneity of injection therapies and reported scores.

### Risk of Bias and Quality of Evidence

**Fig. 5:**
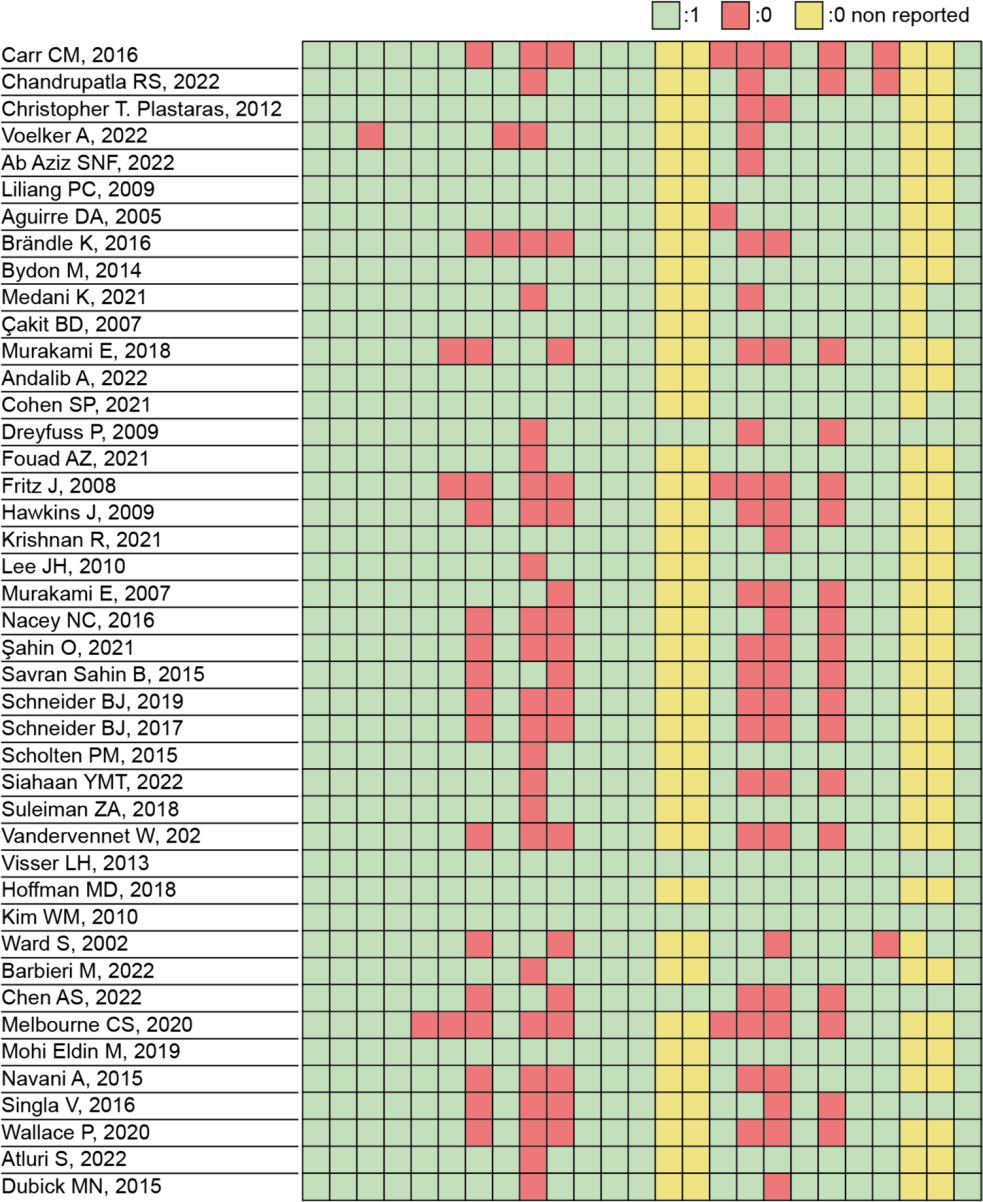
Downs and Black’s checklist

The Downs and Black’s checklist for assessing the risk of bias gives to the studies an excellent ranking for scores ≥26, good for scores from 20 to 25, fair for scores between 15 and 19, and poor for scores ≤14 points [19]. According to these criteria, 2 studies were classified as excellent, 23 as good, 17 as fair, and 1 as poor (Fig. 5). Among the studies meta-analyzed, 2 were classified as excellent and 11 as good. The paucity of blinded and randomized studies, and the lack of probability values and random variability lowered the quality of the enrolled studies.

## Discussion

The main finding of this systematic review and meta-analysis is that corticosteroids are the most documented injective approach to treat SIJ pain, with studies in the last 2 decades showing both safety and effectiveness. More recently, an increasing number of studies explores other treatment alternatives, with PRP showing promise to address SIJ patients.

The corticosteroids’ anti-inflammatory action is used to reduce pain in many clinical conditions, such as osteoarthritis, synovitis, tendinitis, tenosynovitis, and others [10]. Analogously, corticosteroids are the most commonly applied injective treatment for SIJ, aiming to provide pain relief by relying on the pleiotropic effects on the inflammatory pathways: downregulating pro-inflammatory cytokines and chemokines, and upregulating the production of anti-inflammatory genes [28]. Among the different available corticosteroids, triamcinolone acetate, methylprednisolone, betamethasone, and dexamethasone have all been used, since no agreement has been reached on the most effective product. In particular, this systematic review identified only one study comparing the efficacy between two steroids [25]. In the recent study of Krishnan et al., the comparison of 23 patients treated with methylprednisolone and 81 receiving one triamcinolone acetate injection showed overall comparable results at 1 month. Unfortunately, beside this comparative non-randomized study limited to a short-term evaluation, no other data are available in the current literature to guide the choice of the most effective product to address SIJ pain.

A consensus was not reached also regarding the most effective approach. Among the studies included in the systematic review, only two compared the intraarticular or periarticular approach using steroids or anesthetic [23, 24]. The study investigating the effect of anesthetic periarticular injections suggests this approach as first line for a major effectiveness [23]. The more recent study conducted by Nacey et al. using steroid injections confirmed the benefits of the easier periarticular approach. A recent systematic review suggested a trend through the years in favor of a combined approach to lead to better results [29], even though no comparative studies were included in that literature analysis. In fact, among the included articles, a combined approach was tested only in steroid injections or human grow hormone injections [15, 30–32] case series, confirming the promising results of targeting both joint and periarticular tissues. To deliver the injected product to the target area, the literature also suggests the use of a radiological guidance, being a blind injection effective only in 22% of the procedures [10]. While no consensus has been reached on the most suitable radiological guidance [33, 34], fluoroscopy is the oldest and most documented approach. When an intraarticular radiological-guided injection approach is chosen, the literature suggests performing the injection in the lower third of the joint [35]. However, more recently ultrasound is gaining attention as a valid alternative to fluoroscopically and TC-guided injections, with similar treatment effect but lower radiations to the patients and to the practitioners, as well as for the capacity to avoid critical vessel injuries [10].

Overall, no major complications were reported in this systematic review, even though the literature largely documented the potential drawbacks of intraarticular steroid and anesthetic injections, with the risk of inducing local chondrolysis and osteoporosis, and, in case of surgery, of increased post-operative pain scores [36–39]. Local corticosteroids injections can lead also to systemic consequences by causing immunosuppression: infection diseases, Cushing’s syndrome, weight gain, fluid retention, mood disturbances, and gastrointestinal upset [28]. The importance of considering the potential complications of corticosteroid is underlined by the fact that most patients need 2 or 3 steroid injections to see benefits, and more injections can be performed through years exposing patients to the potential corticosteroids side effects [28]. In this review, the larger group of patients was 40–60 years old, a relatively young age distribution predisposing to the need for repeated procedures over the years, ultimately leading to increased risks of side effects. Among the included studies, only three have a follow-up longer than 1 year, making it difficult to properly document the risk of repeated injections. Among the population included in the review treated with steroid injections, the majority presented a high BMI, a further indicator of the possibility of systemic diseases such as diabetes, which make steroid treatment contraindicated. While no sub-analysis could be performed to stratify the risks of SIJ injections, an overall low complications rate was found, although the significant rate of failures of steroid injection (26%) supports the need to identify alternative treatment options.

Prolotherapy was proposed to address SIJ pain. This procedure involves an injection of an irritating substances in a damaged zone such as osmotic agents, or chemotactic agents. The irritation induces an influx of inflammatory cells, which can ultimately lead to a healing response and a tissue repair [28]. Notably, one article reporting the worsening of outcomes was a dextrose retrospective study [40], while one of the articles reporting the best improvement was a botulinum case series [13]. The broad range of results warrants further investigation, also considering that a meta-analysis of these studies was not possible due to the variety of injection therapies and scores used.

More recently, PRP gained attention for its use in different joints as an alternative to steroid injections [41–43]. PRP is an autologous sources of growth factors and biomolecules released by platelet degranulation. Platelet alpha-granules release fibroblast growth factors, transforming growth factors beta-1, platelet-derived growth factors, and platelet-derived angiogenesis factors. Platelets release also fibronectin, vitronectin, and sphingosine 1-phosphate. All these molecules can accelerate tissue healing [28]. Different preparations of PRP have been tested, but no sufficient data have been published to clearly demonstrate which product is most suitable for this treatment indication [27, 44]. Despite the paucity of data, it was possible to compare the pain outcomes after infiltrative therapy with corticosteroids or PRP. Patients treated with PRP are usually younger and have lower BMIs than those in the steroid group. Young and fit patients are more difficult to treat due to high functional request and the need for long-lasting results [16, 45]. Accordingly, the overall different indication observed further impairs a comparison of the benefit documented with the two injective approaches. PRP is a relatively new treatment for SIJ pain, and the efficacy of PRP injections is still uncertain due to the lack of consistent literature and of randomized controlled trials. The American Society of Interventional Pain Physician (ASIPP) classified the PRP SIJ injections as level IV evidence [46] due to a lack of evidence. Nonetheless, this systematic review underlined that adverse events occurred with PRP injections were all minor events, such as post-injective pain, vasovagal reactions, and stiffness, and the clinical improvement was significant, thus supporting further research into this biological treatment approach.

The limitations of this systematic review and meta-analysis reflect those of the analyzed literature, which presented highly heterogeneous studies. The difference in the used scores and follow-up times evaluated makes it difficult to directly compare cohort studies, even when they use the same treatments. The lack of consensus on the best steroid to use and the best PRP preparation adds confusion with several products tested. Moreover, outcomes were never reported based on the sex of the studied population. This is remarkable because males and females have different SIJ anatomy, different inflammatory responses, and comorbidities. Female sacral cartilage is thicker, while cortical bone is thinner, and female have higher mobility and more pelvis ligaments strains compared to men. Also the influence of hormones in females is significant for laxity and pain of SIJ [47]. Thus, more data with a gender-based focus are needed to better explore potential and indications of the different injective treatments for SIJ. Finally, associated treatments like oral drugs and physical therapies have been scarcely reported, all factors that could influence the overall response to the applied injectable treatment. Despite the aforementioned limitations, this systematic review and meta-analysis offered important indications on the advantages and disadvantages of the analyzed injective strategies. The results of this study highlight the potential of different injection therapies, which could be of clinical relevance for physicians managing SIJ patients, as well as for researchers planning future studies to optimize SIJ treatment.

Corticosteroids are the most documented injective approach to treat SIJ pain, with studies in the last 2 decades showing an overall safety and effectiveness. Still, the high number of failures underlined by some studies suggest the need for alternative procedures. An increasing number of studies shows promise for PRP, but the limitations of the current literature do not allow to clearly define the most suitable injective approach, and further studies are needed to identify the best injective treatment for SIJ patients.

## Data Availability

Not applicable.
